# Paraxial Ocular Measurements and Entries in Spectral and Modal Matrices: Analogy and Application

**DOI:** 10.1155/2014/950290

**Published:** 2014-06-19

**Authors:** Herven Abelman, Shirley Abelman

**Affiliations:** ^1^Biomedical Engineering Research Group, School of Electrical and Information Engineering, University of the Witwatersrand, Johannesburg, Private Bag 3, Wits 2050, South Africa; ^2^School of Computational and Applied Mathematics, University of the Witwatersrand, Johannesburg, Private Bag 3, Wits 2050, South Africa

## Abstract

Lensometers and keratometers yield powers along perpendicular meridians even if the principal meridians of the lens and the cornea are oblique. From each such instrument, multiple raw data represented on optical crosses require conversion to determine elementary statistics. Calculations for research decisions need to be authentic. Principles common to meridians generalize formulaic methods for oblique meridians. Like a lens or a cornea, matrix latent quantities are represented on a matrix cross. Our problem is to determine the matrix whose cross represents quantities on the optical cross. All measurements on an optical cross that include corneal and lens powers and oblique meridians can be considered. Once determined, a portfolio of matrix calculations applies and is justified for ophthalmic calculation. Matrices can be unique and, like a cornea before it is measured, contain latent observations. Asymmetric power component matrices quantify a deviation of a corneal surface from smoothness and toricity. Entries may identify those measurements causing irregular astigmatism that may stem from surgical or other external intervention. Irregular astigmatism is detected primarily from significant measurements in the paraxial range. Measurements are assimilated with matrix factors in a holistic way in order to support choices with calculations and statistics.

## 1. Introduction

Paraxial ocular measurements take on a format with standardized, clearly formulated rules for processing when represented by eigenvalues and eigenvectors of some array. Distinct principal powers and meridians, real or complex conjugate, are used to obtain a unique real array holistically. The reverse process determines eigenvalues and eigenvectors of a given dioptric power array that represent principal powers and meridians. From the equations that ensure unique arrays we show that coincident real principal powers can yield multiple arrays. On the small zone of the cornea sampled with a keratometer near an entrance pupil, we model the primary contribution to irregular astigmatism. We use matrix properties to show unequivocally how an angle not 90° and not 0° between meridians and the length of the interval of Sturm in dioptres are paraxial contributors to this effect. Among other measurements, typical keratometric data are placed in a format to determine relevant statistics (impossible in the raw form). The paper is set within the context of linear optics and concerns only first-order effects.

**Figure 1:**
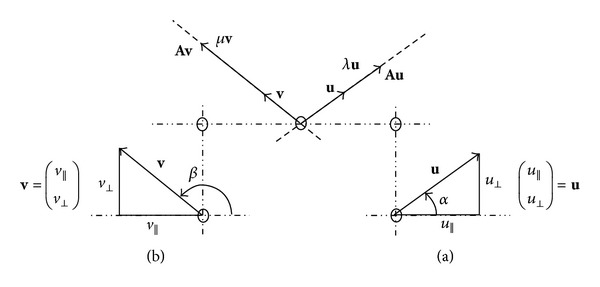
Eigenvectors **u** and **v** of **A** multiplied by **A** yield **A** **u**  and **A** **v** that are parallel to **u** and to **v**, respectively since *λ* **u** = **A** **u** and *μ* **v** = **A** **v**. The upper central figure is analogous to an optical cross if we represent principal powers of a lens or corneal surface by *λ* and *μ* and the directions of principal meridians by **u** and **v**, respectively. Components in $u=\left(\begin{array}{c} u_{\left|\right|}\\ u_{⊥} \end{array}\right)$ and $v=\left(\begin{array}{c} v_{\left|\right|}\\ v_{⊥} \end{array}\right)$ placed along meridians are processed to yield angles *α* and *β* at O in (a) and (b). The plane of the figure is tangent to an optical surface at its vertex O.

An effective difference in the power along principal meridians of the refracting media will be detected as astigmatism in an eye. The refraction may be compensated along meridians of greater and lesser power, at right angles to one another for continuous surfaces. Powers along other meridians are then simultaneously offset except in cases where the continuity of the surface element is compromised. Greatest and least powers, called principal powers (initially assumed to be distinct) and the directions of their meridians from, say, a keratometer, are analogous to eigenvalues and eigenvectors of data arrays [1], respectively, (see numerical Example 2 and the optical cross in Figure 1). Matrices and their elements may be used to monitor continuity of paraxial surface elements and disclose information for all meridians. Matrices are compact and complete and represent heterogeneity particularly for oblique meridians on the cornea. Linear algebra in standard texts [2–4] is applied to fields that include medical imaging, engineering and in ophthalmic literature [1, 5–8], where eigenvalues and eigenvectors often represent observable and measureable quantities.

**Figure 2:**
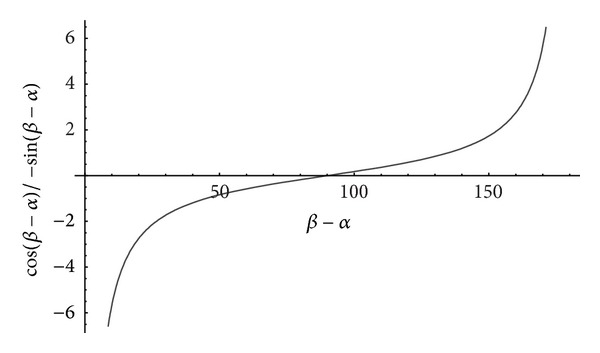
Graph of the angle *β* − *α* in degrees between the principal meridians and the trigonometric factor in irregular astigmatism *F*_*L*_ in (8). The curve crosses the horizontal axis at 90°  (*F*_*L*_ = 0 D) and is an uneven function with respect to 90°: −*F*_*L*_(90 − *θ*) = *F*_*L*_(90 + *θ*). Above 90° the trigonometric factor is positive.

Principal meridians at right angles produce regular astigmatism that is correctable with a spherocylindrical lens [9, 10]. Principal meridians of highly toric surfaces separated by an angle significantly less or greater than 90° may cause irregular astigmatism to manifest. Careful measurements made separately for the two principal meridians are not usually exactly perpendicular. See Figure 2. This may not imply significant irregular astigmatism. Measurement errors are likely to be more significant than irregular astigmatism for spherocylindrical surfaces. Patients with irregular astigmatism may measure a loss of spectacle-corrected visual acuity. In keratometry distorted focused mires are among the other simpler signs. In addition to the orientation of the principal meridians, different parts of the same meridian can have different curvatures across an entrance pupil of the eye [11]. Thus irregular astigmatism may be attributed to patients with substantial irregular corneal surface elements. Significant irregular astigmatism is uncommon and could be related to a scarred cornea, pterygium [12], and surgical procedures. Eigenvectors and eigenvalues vary from surface element to element across an entrance pupil.

Spherocylindrical lenses, perpendicular meridians, and symmetric matrices [5, 13] facilitate calculations of prismatic effect as well as the power of obliquely crossed lenses. In the obliquely crossed lens problem, the sphere, cylinder, and axis of the equivalent lens were extracted (see numerical Example 2 and Figure 1) from a matrix [5, 13] with a particular modus operandi that made use of the invariance of the trace and determinant of the power matrix similarity transform. In previous work [14] each principal meridian on the front surface of a cornea was independently aligned with the focused mires of a keratometer. In this paper, we integrate optical crosses and labels with the geometrical picture of eigenvectors and eigenvalues of matrices in Figure 1, whenever we place principal clinical observations in spectral matrices and coordinates of the axes of an optical cross in corresponding modal matrices. In a majority of cases, the integration allows modal and spectral matrices to be multiplied and converted holistically to the power matrix. Characteristic equations and their linearity, partially exploited in previous work [14], explicitly generalize decisions on the uniqueness of the matrix, how modal and spectral matrices are compromised and why one can obtain many matrices. We start from a geometric picture in a general way to obtain a power matrix. We refer to [15] and references cited therein.

Measurements of paraxial phenomena along principal meridians, separate or coincident, (or selected complex conjugate ones) and their analogues generated from a matrix in Figure 1 are considered exclusively. Matrices quantify heterogeneity in the physiological optics of the local eye and vision correction. Among measurements, this common thread serves to determine their matrices that can separate regular from irregular astigmatism. Matrices can now be determined for principal meridians of arbitrary location for an astigmatic eye and various medical and engineering fields. We show that the initial contributor to irregular astigmatism on a cornea is principal meridians that are significantly nonperpendicular and which specialist equipment is probably capable of resolving in the best way. This paper justifies calculations with ophthalmic variables and measurements, characteristic of some matrix, in the paraxial domain. Scalar data sets emanating from computer software in specialist clinics sample global corneal properties.

In this paper, the power matrix from meridians of arbitrary orientation is written as a sum of a symmetric and an antisymmetric matrix [2, 16, 17]. The symmetric matrix represents a paraxial power component with perpendicular meridians for the continuous surface portion. The asymmetric component independently represents the deviation from toricity or smoothness of a cornea following, for example, surgical intervention or abnormal growth owing to a dusty windy environment. From this we confirm that distinct keratometer powers along significantly oblique meridians contribute to irregular astigmatism. Corneal power for rectangular meridians is augmented by paraxial asymmetric power in (7).

Gaussian optics is often referred to as the optics of perfect optical systems. First-order equations can be derived by reducing the exact trigonometrical expressions for ray paths to the limit when the angles and ray heights involved approach zero. These equations are completely accurate for a small region, known as the* paraxial* region. A well-corrected optical system will follow the first-order expressions almost exactly. The first-order image positions and sizes provide a convenient reference from which to measure departures from perfection. In addition, the paraxial expressions are linear and are much easier to use than the trigonometrical equations [18]. Figure 1 represents arbitrarily chosen matrix characteristics on the upper half-plane on the page tangent to and symbolic of a surface (vertex O) where the refraction of rays is thought to take place.

## 2. Method

Measurements are formatted and tools developed to make valid calculations. A paraxial element of a cornea or an ophthalmic lens, appropriately stopped, and a matrix both have latent characteristics. We reconcile measurements in physiological optics with matrix characteristics in linear algebra so that calculations may be made. The outputs from a keratometer or a lensometer are very closely related to eigenvalues and eigenvectors of an appropriate matrix. An entire portfolio of matrix calculations is fully justified and applies to ophthalmic quantities. Principal powers and principal meridians are associated with eigenvalues and eigenvectors and determine a matrix.

Let 2 × 2 matrix **A** multiply nonzero vectors **u** and **v**; then new vectors **A** **u** and **A** **v** result. If the vector **A** **u** has the same direction as **u** (and **A** **v** has the same direction as **v** but different from **u**), then **u** and **v** are called eigenvectors of **A**. They satisfy [2, 3]

(1) $$\begin{array}{c} Au=\lambda u+0v,\\ Av=0u+\mu v, \end{array}$$

where *λ* and *μ* are real scalars called eigenvalues of real **A** associated with real **u** and **v**, respectively. When **A** multiplies vectors different from **u** and **v** on the left, coefficients of both **u** and **v** on the right in (1) are both nonzero and vectors different from **u** and **v** on the left are not characteristic of **A**. This means that a toric ophthalmic surface has but two principal meridians and any other meridians are not principal in nature. Vectors **u**, *λ* **u**, and **A** **u** have the same direction and independently **v**, *μ* **v**, and **A** **v** all point in another (includes antiparallel) direction. These vectors satisfy (1) and are represented in Figure 1. Eigenpairs of **A** are (*λ*, **u**) and (*μ*, **v**). Vectors **A** **u** and **A** **v** can be obtained from the much simpler positive, negative, or zero scalar multiplication of eigenvectors **u** and **v** by associated values *λ* ≠ 0 ≠ *μ*. If the eigenvalues of a matrix are distinct, then the associated eigenvectors are linearly independent. The directions of the meridians in Figure 1 are the horizontal and vertical components in the columns of $u=\left(\begin{array}{c} u_{\left|\right|}\\ u_{⊥} \end{array}\right)$ and $v=\left(\begin{array}{c} v_{\left|\right|}\\ v_{⊥} \end{array}\right)$ .

We now facilitate calculations with data on an optical cross. Using matrix multiplication, we combine vector equations (1) into a single matrix equation:

(2) $$\begin{array}{c} A\left(u\;v\right)=\left(u\;v\right)\left(\begin{array}{cc} \lambda & 0\\ 0 & \mu \end{array}\right). \end{array}$$

Matrix **A** operates on an array (**u** **v**) of eigenvector columns. Since (*λ* ≠ 0 ≠ *μ*), the array (**u** **v**) is nonsingular and we write

(3) $$\begin{array}{c} A=\left(u\;v\right)\left(\begin{array}{cc} \lambda & 0\\ 0 & \mu \end{array}\right){\left(u\;v\right)}^{-1}{\left(\begin{array}{cc} u & v \end{array}\right)}^{}. \end{array}$$

Matrix **A** is expressed as a product of matrices multiplied in the order shown that contain measurements on a power cross and one factor matrix is the inverse of the other. The columns **u** and **v** in the matrix (**u** **v**) define the arms of the optical cross or the principal meridians as in Figure 1. Eigenvector matrix (**u** **v**) is also called a modal matrix [4]. In the first column in the central matrix *λ* is the eigenvalue that is associated with the column **u** in the matrix (**u** **v**). The central matrix in (3), with principal powers on the diagonal, is called an eigenvalue matrix or spectral matrix.

Equation (3) is known as* eigenvalue decomposition *or* matrix similarity transform *[2, 14]. It is valid since **u** and **v** are independent [2] for *λ* ≠ 0 ≠ *μ* and whether **u** and **v** are perpendicular or not. Vectors **u** and **v** in Figure 1 are analogous to the directions of principal meridians. These may be meridians of the anterior corneal surface for which powers and curvatures are maxima and minima. They could also be directions for which radii of curvature are extreme. These corresponding principal quantities, all determined with procedures or instrumentation, are represented by eigenvalues *λ*, *μ* of **A**. Matrices **A** with *λ* ≠ 0 ≠ *μ* are unique and nonsingular but later, in this paper, we discuss the effect of relaxing this constraint on uniqueness. Application to an anterior cornea is now discussed and its irregular paraxial surface is quantified.

A generalized corneal reading [19] is

(4) $$\begin{array}{c} \lambda \left\{\alpha \right\}\mu \left\{\beta \right\}, \end{array}$$

where distinct principal powers *λ* and *μ* are associated with their respective meridians at angles *α* and *β* directed along units **u** and **v** on an optical cross like that in Figure 1, where O is at an entrance pupil centre. The components *u*_||_, *u*_⊥_ and *v*_||_, *v*_⊥_ called direction cosines of **u** and **v** along principal meridians are

(5) $$\begin{array}{c} \left(u\;v\right)=\left(\begin{array}{cc} \cos\alpha & \cos\beta \\ \sin\alpha & \sin\beta \end{array}\right) \end{array}$$

which has no particular units. In Figure 1 angles shown are restricted to the range (0, 180°] which make this representation of the principal meridians as vectors unique (*β* ≠ *α*). Together with (5), Equation (3) becomes

(6) A=1sin(β−α)(cos⁡αcos⁡βsinαsinβ) ×(λ00μ)(sinβ−cos⁡β−sinαcos⁡α).

Given principal powers and corresponding meridians, the power matrix was obtained by different calculations for each of the four entries [19]. Principal data in the spectral matrix and column vectors along corresponding meridians in the modal matrix (**u** **v**) and these factors are multiplied as in (6) to yield a power **A**. For meridians to coincide *β* = *α* and an essential singularity exists in (6). The dependence of **u** and **v**  (*β* = *α*) clearly implies that a matrix (ces) in (1) has reduced rank compared to their order. Thus **A** is only unique when *β* ≠ *α*  (*λ* ≠ 0 ≠ *μ*). This aspect is considered later.

We denote the transpose of **A** by **A** ^*T*^. We multiply the matrices in (6) and write the product **A** as a sum of two matrices [2]: **A** = (1/2)(**A** + **A** ^*T*^)+(1/2)(**A** − **A** ^*T*^), where

(7) 12(A+AT)=1sin(α−β) ×(μcos⁡βsinα−λcos⁡αsinβ(λ−μ)cos⁡(α+β)2(λ−μ)cos⁡(α+β)2λcos⁡βsinα−μcos⁡αsinβ),12(A−AT)=FL(01−10)

with *F*_*L*_ given by

(8) $$\begin{array}{c} F_{L}=\frac{\left(\lambda -\mu \right)\cos\left(\alpha -\beta \right)}{2\sin\left(\alpha -\beta \right)} \end{array}$$

which is a symbol in common with (17) in [16], *β* ≠ *α*. Astigmatism for the anterior cornea is regular when perpendicular principal meridians emerge as they do when *F*_*L*_ is zero and **A** is symmetric. On irregular astigmatism, we plot the trigonometric factor in (8) which is seen not to exist when *β* = *α*.

The curve shows greater sensitivity to change for angles less than approximately 40° and greater than approximately 140°. In the approximate range from 60° to 120° the trigonometric factor attenuates *F*_*L*_. Outside this range the trigonometric factor amplifies *F*_*L*_. When *β* → *α* irregular astigmatism occurs and *F*_*L*_ → −*∞*.

The matrix $\left(\begin{array}{cc} 0 & 1\\ -1 & 0 \end{array}\right)$ expresses irregular astigmatism of a unique amount (*β* ≠ *α*) given in (8) on an irregular anterior cornea confirmed from the eigenvectors belonging to **A**. As in [9, 10, 20], (8) ascribes irregular astigmatism to distinct principal powers along meridians that are significantly off-perpendicular or off-parallel detected on a small zone on a cornea. One needs to sample larger corneal zones to detect the other contributors. Figure 2 shows that asymmetric power is zero when *β* − *α* = 90°. Equations (7) show that regular astigmatism can exist whether irregular (asymmetric) astigmatism is zero (meridians are perpendicular) or not.

Where principal meridians are not 90° apart, associated loss of spectacle-corrected vision represents one of the most serious and frequent complications of corneal refractive surgery [10]. If *λ* = *μ*, regular astigmatism is zero and the irregular astigmatism equation (8) is zero irrespective of meridian location. The matrix in (7) applies and becomes

(9) $$\begin{array}{c} A=\lambda \left(\begin{array}{cc} 1 & 0\\ 0 & 1 \end{array}\right) \end{array}$$

which describes a spherical paraxial surface. Equation (8) is valid for all surfaces, where *λ* − *μ* is the length in dioptre of the interval of Sturm on a number line of principal powers and (*β* − *α*) is the angle between principal meridians. First-order optics and paraxial analysis are tools with which we consider small corneal zones for irregular astigmatism and its simplest cause. For a given surface, the angle (*β* − *α*) remains unchanged as the surface rotates; *α* and *β* are now mutually dependent and as the surface rotates, *α* + *β* in the symmetric part in (7) assumes new values.

Suppose that *β* = *α* + 90°, then the second matrix in (7) is a null matrix and the first matrix for perpendicular meridians becomes

(10) $$\begin{array}{c} A=\left(\begin{array}{cc} \frac{1}{2}\left(\lambda +\mu +\left(\lambda -\mu \right)\cos2\alpha \right) & \left(\lambda -\mu \right)\cos\alpha \sin\alpha \\ \left(\lambda -\mu \right)\cos\alpha \sin\alpha & \frac{1}{2}\left(\lambda +\mu +\left(-\lambda +\mu \right)\cos2\alpha \right) \end{array}\right). \end{array}$$

Driven by redness, irritation, and tearing in hot, dry sunny environments, a pterygium can grow on the conjunctiva onto the clear cornea and changes in axis and power of astigmatism may be detected. Depending on the progression of growth, the pterygium may cause the anterior surface to vary [12] irregularly of which the irregular astigmatism in (8) is a processed measure. Topography should confirm the nature of the local or general variation of the corneal surface and irregular astigmatism should be diagnosed (outside of the paraxial) by different segments of the same meridian having different powers [9, 10, 20]. When the cornea heals after refractive laser surgery and leaves a discontinuous anterior surface, irregular astigmatism may manifest. An antisymmetric dioptric power matrix quantifies the power of the significantly uneven surface created by the laser. A hypothetical rough surface with power quantified in exactly the same way is described in [21].

Wherever the spectral matrix is diagonal, *λ* ≠ *μ* ≠ 0, we ensure (i) the independence of the **u** and **v**, (ii) det⁡⁡(**u** **v**) ≠ 0, (iii) the uniqueness of matrix **A**, and (iv) det⁡(**A**) ≠ 0. Transpose the system of (1) and (3) and let the unknown $A=\left(\begin{array}{cc} a_{11} & a_{12}\\ a_{21} & a_{22} \end{array}\right)$ . Then

(11) $$\begin{array}{c} \left(\begin{array}{cc} u_{\left|\right|} & u_{⊥}\\ v_{\left|\right|} & v_{⊥} \end{array}\right)\left(\begin{array}{cc} a_{11} & a_{21}\\ a_{12} & a_{22} \end{array}\right)=\left(\begin{array}{cc} \lambda & 0\\ 0 & \mu \end{array}\right)\left(\begin{array}{cc} u_{\left|\right|} & u_{⊥}\\ v_{\left|\right|} & v_{⊥} \end{array}\right), \end{array}$$

where the matrices (**u** **v**)^*T*^ and diag⁡(*λ*, *μ*) known from clinical measurements are full rank.

Suppose *λ* ≠ 0 ≠ *μ* is no longer valid. Spectral matrix in (11) becomes a scalar matrix. Rank of (**u** **v**)^*T*^ is now reduced and **u** + *s* **v** = 0 for scalar *s* ≠ 0. det⁡(**A**) = 0 when  *λ* = 0. Equation (11) becomes the identical, equivalent equation

(12) $$\begin{array}{c} -sv_{\left|\right|}\left(\begin{array}{c} a_{11}-\lambda \\ a_{21} \end{array}\right)-sv_{⊥}\left(\begin{array}{c} a_{12}\\ a_{22}-\lambda \end{array}\right)=0,\\ v_{\left|\right|}\left(\begin{array}{c} a_{11}-\lambda \\ a_{21} \end{array}\right)+v_{⊥}\left(\begin{array}{c} a_{12}\\ a_{22}-\lambda \end{array}\right)=0. \end{array}$$

A single equation (12) in two unknown column vectors has multiple solutions for **A**. Denominators in components in [14] and entries of antisymmetric **A** are zero in (7) and (8) when **u** + *s* **v** = 0. One may consider the generalized inverse of (−*s* **v** **v**)^*T*^ or determine eigenvectors for powers of **A**, called power vectors; they form a complete basis for a matrix in which there are fewer linearly independent eigenvectors than eigenvalues [5]. Particular ambiguous 2 × 2 real matrices with parallel eigenvectors predicted in (12) may be found in [14, 19].

Given a vector **x** with complex entries. Then $\overset{-}{x}$ denotes the complex conjugate of **x**. Suppose that 2 × 2 real **A** can multiply **x**. Then

(13) $$\begin{array}{c} \bar{Ax}=\overset{-}{A}\overset{-}{x}=A\overset{-}{x}. \end{array}$$

If *λ* is an eigenvalue of **A** and **x** is a corresponding eigenvector, then

(14) $$\begin{array}{c} A\overset{-}{x}=\bar{Ax}=\bar{\lambda x}=\overset{-}{\lambda }\overset{-}{x}. \end{array}$$

Hence $\overset{-}{\lambda }$ is also an eigenvalue of **A**, with $\overset{-}{x}$ a corresponding eigenvector. If a real 2 × 2 matrix has eigenvalues that are complex, the corresponding eigenvectors are also complex and the eigenpairs are complex conjugates.

Suppose that *λ* = *a* − *bi* and **x** = **u** − **v** *i*. Then

(15) Ax=Au−Avi=(a−bi)(u−vi)=au−bv−(bu+av)i.

The other eigenvalue is $\overset{-}{\lambda }=a+bi$ with eigenvector $\overset{-}{x}=u+vi$ .

Consider

(16) $$\begin{array}{c} x+\overset{-}{x}=2u,\;\;x-\overset{-}{x}=-2vi,\\ A\overset{-}{x}=Au+Avi=\left(a+bi\right)\left(u+vi\right)=au-bv+\left(bu+av\right)i, \end{array}$$

(17) A(x+x−)=2Au=2(au−bv)so that  Au=au−bv,A(x−x−)=−2Avi=−2(bu+av)iso that  Av=bu+av.

Independent **u**, **v** and scalars *a*, *b* are real. When *b* = 0, (17) reduces to (1) when eigenvalues are equal. In numerical Example 3 we explain how one complex eigenpair (*λ*, **x**) produces real matrix factors **C** and **P** of a real dioptric power matrix **A** using the theory above.

When the matrix on the right hand side in (7), called **L**, premultiplies real vectors, products are all vectors rotated clockwise and perpendicular to the real vectors. Products with eigenvectors are parallel to the complex vectors mentioned with complex eigenvalues below. The characteristic equation of **L** is *λ* ^2^ + 1 = 0. Spectral matrix of the antisymmetric **L** is $\left(\begin{array}{cc} i & 0\\ 0 & -i \end{array}\right)$ and its orthogonal modal matrix is $\left(1/\sqrt{2}\right)\left(\begin{array}{cc} -i & i\\ 1 & 1 \end{array}\right)$ . From this matrix, the meridians at an angle *w* satisfy tan *w* = *i* or −*i* which has no real or complex solution. Thus meridians where principal powers are *λ*_1_ = *i* and *λ*_2_ = −*i* do not exist.

## 3. Examples

We now illustrate the concepts considered in our paper by discussing three numerical examples.

**Example 1 .**

Suppose that a lens surface has a principal meridian at an angle whose tangent is 12/5 in Figure 1(a) and the other principal meridian is at an angle whose tangent is 8/–15 in Figure 1(b) with powers 4 D and –7 D, respectively. Determine the power matrix of the surface using the eigenvalue decomposition.

*Solution*. The meridians are not perpendicular ((12/5) × (8/−15) *≠* −1) so that both meridians and their principal powers specify the surface. The components of the first meridian in Figure 1(a) are $\left(\begin{array}{c} u_{\left|\right|}\\ u_{⊥} \end{array}\right)=\left(\begin{array}{c} 5/13\\ 12/13 \end{array}\right)$ and those of the second meridian in Figure 1(b) $\left(\begin{array}{c} v_{\left|\right|}\\ v_{⊥} \end{array}\right)=\left(\begin{array}{c} -15/17\\ 8/17 \end{array}\right)$ . We place these in the eigenvector matrix (**u** **v**) in the order in which they were determined. $(u\;v)=\left(\begin{array}{cc} 5/13 & -15/17\\ 12/13 & 8/17 \end{array}\right)$ and ${\left(u\;v\right)}^{-1}=\left(\begin{array}{cc} 26/55 & 39/44\\ -51/55 & 17/44 \end{array}\right)$ . The eigenvalue matrix with principal powers in the order of the meridians corresponding in (**u** **v**) is

(18) $$\begin{array}{c} \left(\begin{array}{cc} \lambda & 0\\ 0 & \mu \end{array}\right)=\left(\begin{array}{cc} 4 & 0\\ 0 & -7 \end{array}\right)\;\text{D}. \end{array}$$

Substituting into (3), we obtain

(19) A=(513−15171213817)(400−7)(26553944−51551744)=(−51542452)D,

which is the sum of the two matrices as in (7)

(20) $$\begin{array}{c} A=\left(\begin{array}{cc} -5 & \frac{171}{40}\\ \frac{171}{40} & 2 \end{array}\right)+\left(\begin{array}{cc} 0 & -\frac{21}{40}\\ \frac{21}{40} & 0 \end{array}\right)\;\text{D}. \end{array}$$

Dioptric power **A** of the surface has been expressed as a sum of symmetric and antisymmetric matrices. The symmetric matrix includes regular astigmatism. The nonzero entries in the antisymmetric matrix indicate that the principal meridians of the lens surface are not perpendicular. This is not a spherocylindrical surface. We identify a surface with antisymmetric power –21/40 D which may compensate for irregular corneal astigmatism [22]. It is a component that most lens manufacturers will endeavour to minimize by making principal meridians perpendicular as it is believed to divert illumination energy away from the focal point in the eye.

**Example 2 .**

This example quantifies the degree of irregularity of the paraxial corneal surface.

Suppose that we have the power matrix

(21) $$\begin{array}{c} A=\left(\begin{array}{cc} 44-\sqrt{3} & 1\\ 2\sqrt{3}-3 & 42+\sqrt{3} \end{array}\right)\;\text{D} \end{array}$$

of a corneal surface near the entrance pupil. Where on this cornea are the principal meridians and what are the corresponding principal powers? To answer this we determine two eigenvectors and corresponding eigenvalues or measure the cornea on a keratometer.

*Solution (see [2]).* Principal meridians along eigenvectors **u**, **v** in Figure 1 and corresponding scalars *λ*, *μ* that represent principal powers can be measured with a keratometer. Eigenvalues and corresponding **u** exist for which **A** **u** = *λ* **u**.

Rewrite this equation as (**A** − *λ* **I**)**u** = 0, where **I** is the identity matrix $\left(\begin{array}{cc} 1 & 0\\ 0 & 1 \end{array}\right)$ .

Consider

(22) (A−λI)u=((44−3123−342+3)−(λ00λ))u=(44−3−λ123−342+3−λ)u=(00).

The determinant of coefficient matrix (**A** − *λ* **I**) is zero when

(23) $$\begin{array}{c} \mu =42,\;\;\lambda =44 \end{array}$$

and the coefficient matrix is singular. The principal meridian **u**, where the principal power is 44 D, is any multiple of $u=\left(\begin{array}{c} 1\\ \sqrt{3} \end{array}\right)$ and is plotted in Figure 1(a). Our meridian is then parallel to a unit vector $\left(\begin{array}{c} 1/2\\ \sqrt{3}/2 \end{array}\right)$ with *α* = arctan√3 = 60°, where *α* is the angle seen in Figure 1(a). The meridian, where the principal power is 42 D, is along a vector that is any multiple of $v=\left(\begin{array}{c} -2-\sqrt{3}\\ 1 \end{array}\right)$ . This meridian is plotted in Figure 1(b), where *β* = arctan (−2 + √3) = 165°. Thus a power “cross” along meridians is not perpendicular as shown in Figure 1(b).

Using (3), factors of $A=\left(\begin{array}{cc} 44-\sqrt{3} & 1\\ 2\sqrt{3}-3 & 42+\sqrt{3} \end{array}\right)\;\text{D}$ are

(24) $$\begin{array}{c} \left(\begin{array}{cc} \cos60^{{}^{\circ}} & \cos165^{{}^{\circ}}\\ \sin60^{{}^{\circ}} & \sin165^{{}^{\circ}} \end{array}\right)\left(\begin{array}{cc} 44 & 0\\ 0 & 42 \end{array}\right){\left(\begin{array}{cc} \cos60^{{}^{\circ}} & \cos165^{{}^{\circ}}\\ \sin60^{{}^{\circ}} & \sin165^{{}^{\circ}} \end{array}\right)}^{-1}\;\text{D} \end{array}$$

or on a power cross 44.0 {60} 42.0 {165}. The matrix factors of **A** contain principal powers as measured with a keratometer and corresponding meridians are defined in the columns of trigonometric functions whose arguments are the angles of principal meridians. As in (7)

(25) $$\begin{array}{c} A=\left(\begin{array}{cc} 44-\sqrt{3} & -1+\sqrt{3}\\ -1+\sqrt{3} & 42+\sqrt{3} \end{array}\right)+\left(\begin{array}{cc} 0 & 2-\sqrt{3}\\ -2+\sqrt{3} & 0 \end{array}\right)\;\text{D}. \end{array}$$

The magnitude of irregular astigmatism is |2 − √3|  D.

**Example 3 .**

Assume that some clinical instrument measures principal powers and directions of meridians that are complex eigenvalues and eigenvectors within the context of matrices with real entries. The complex components of eigenvectors alone (real ones are seen in Figure 1 and numerical Example 1) are straightforward, single-valued, and are sufficient here. Suppose that conjugate eigenpairs

(26) λ=4−i,  x=(1−i1),λ−=4+i,  x−=(1+i1)

are given for some real **A**. The real factors of real dioptric power **A** follow.

*Solution*. For the first eigenpair we have [2, page 321]

(27) {a−bi=4−1i}, a=4,  b=1,Re(x)=(11),  Im⁡(x)=(−10).

Arctan (−1/4) and √(4^2^ + 1^2^) are the principal argument and modulus of the complex eigenvalue *λ*. Any vector that matrix **C** operates on rotates this vector through an angle *ϕ*  (tan*ϕ* = −1/4) and scales it by a factor √(4^2^ + 1^2^).

Consider

(28) C=(a−bba)=(4−114),P=(Re(x)Im⁡(x))=(1−110).

According to (17), the real factors that make up a power **A** are **P** **C** **P** ^−1^ and

(29) $$\begin{array}{c} A=\left(\begin{array}{cc} 5 & -2\\ 1 & 3 \end{array}\right)=\left(\begin{array}{cc} 5 & \frac{-1}{2}\\ \frac{-1}{2} & 3 \end{array}\right)-\frac{3}{2}\left(\begin{array}{cc} 0 & 1\\ -1 & 0 \end{array}\right)\;\text{D}, \end{array}$$

say, as with (7). The components in eigenvectors **x** and $\overset{-}{x}$ yield angles *w* for which tan*w* = 1/(1 − *i*) and 1/(1 + *i*) that define meridians. Conjugate angles in radians are (Mathematica)

(30) w=12Arctan 2±{i4} ln⁡5 +πkfor  integers  k=0,±1,±2,….

The angles made by complex eigenvectors are not unique. Principal angles for meridians are the conjugate pair *w* = 31.7 ± 23.0*i* measured in degrees.

## 4. Discussion

Real and imaginary parts of a complex principal power became entries in a scaled rotation matrix. Real distinct principal powers denoted on a power-cross became entries in a diagonal matrix. Coordinates of corresponding meridians, real and complex, were written as corresponding columns of unit vectors (as in Figure 1) in a modal matrix. The mentioned matrices, whose entries can be used to draw Figure 1, are factors of a power matrix. These matrix factors, with their entries from measurements, observations, or postulates, were multiplied as set down in (3), (17). Equation (7) determines the symmetric and antisymmetric dioptric power matrix. We showed how to switch holistically from principal meridional to expressions of dioptric power (in (6)) and back (in detail in numerical Example 2) using the decomposition for dioptric power matrices with real distinct eigenvalues (principal powers) and complex principal powers and meridians with real matrices. The direct and inverse procedures in this paper are attainable from each other and generalize particular content in [5, 13, 14, 19]. We also determined a matrix for principal powers that were equal irrespective of their meridians. Principal meridians that may not necessarily be perpendicular and associated matrices that may not necessarily be symmetric were shown to be unique when principal powers were distinct. Equations which ensured uniqueness had multiple solutions for identical principal powers.

Our methods are applicable to ophthalmic systems that include lenses, surfaces of a lens or cornea, lens effectivity, magnification, and retinoscopy along meridians and various medical and engineering fields, where matrices are generally symmetric and of higher order. Resolution of the power matrix into a sum of symmetric and antisymmetric matrices allowed us to consider a potential deviation of a corneal surface from smoothness and toricity. This, with other signs, may identify the measurements contributing to irregular astigmatism that may be a result of surgical or other external intervention.

Meridians and the dioptric power matrix demonstrated the techniques and three numerical examples yielded results that were generally valid. In the first example two principal powers and corresponding nonperpendicular components of vectors along meridians of a lens surface were given. Eigensystems containing entries modified from the given measurements were multiplied as an eigenvalue decomposition to yield an antisymmetric dioptric power matrix. In the second numerical example, an antisymmetric power matrix was given. Principal powers and corresponding meridians that were not perpendicular (although the methods are valid if meridians are perpendicular) were produced for the surface as the eigenvalues and eigenvectors of the given matrix. Principal meridians and powers are analogous to the eigenvectors and eigenvalues indicated in Figure 1. The given matrix was factorized into modal and spectral matrices containing observable quantities as entries. The irregular power on the cornea was determined. In the third example two complex conjugate eigenvalues and corresponding nonorthogonal complex conjugate eigenvectors of a lens surface with a real matrix were given. An eigenpair, thought to represent observations, was used to determine real factors that were matrices multiplied to yield the real antisymmetric dioptric power matrix that is the same as that from matrix decomposition with complex factors. The matrix is unique as its complex conjugate eigenvalues are always distinct.

## 5. Conclusion

Our paper is fundamental, universal yet paraxial and assimilates measurements with matrices and their factors in a holistic way to support decisions with calculations and statistics. The point of departure is Figure 1 and a set of linear equations (1) whose solution is unique for real distinct meridians. These form the majority of cases for real clinical measurements. A relaxation of these constraints reduces the rank of matrices and compromises uniqueness of the solution of linear equations (1). We have calculated a paraxial contribution to a polynomial expansion of aberrations [23] for irregular astigmatism. A number of everyday phenomena have our method as basis. Its particular innovation and distinction is that irregular astigmatism (a third order aberration) is screened primarily with keratometric measurements. Elegant, costly software and hardware investigations accompanying corneal topography and wavefront sensors should follow on referral [24]. This paper weds arrays with every possible optometric measurement that can be represented on an optical cross and is not specific to corneal or lens powers like previous related work. The paraxial component of power that expresses the deviation from smoothness and toricity following surgical intervention or abnormal corneal growth owing to a dusty windy environment is defined here. Our work forms a solid basis for future work.
